# Clinical data sharing improves quality measurement and patient safety

**DOI:** 10.1093/jamia/ocab039

**Published:** 2021-03-13

**Authors:** John D D’Amore, Laura K McCrary, Jody Denson, Chun Li, Christopher J Vitale, Priyaranjan Tokachichu, Dean F Sittig, Allison B McCoy, Adam Wright

**Affiliations:** 1 Informatics Department, Diameter Health, Farmington, Connecticut, USA; 2 Kansas Health Information Network, Topeka, Kansas, USA; 3 School of Biomedical Informatics, University of Texas Health Science Center at Houston, Houston, Texas, USA; 4 Department of Biomedical Informatics, Vanderbilt University Medical Center, Nashville, Tennessee, USA

**Keywords:** health information interoperability, electronic clinical quality measure (eCQM), Quality Indicators, Health Care, Patient Safety

## Abstract

**Objective:**

Accurate and robust quality measurement is critical to the future of value-based care. Having incomplete information when calculating quality measures can cause inaccuracies in reported patient outcomes. This research examines how quality calculations vary when using data from an individual electronic health record (EHR) and longitudinal data from a health information exchange (HIE) operating as a multisource registry for quality measurement.

**Materials and Methods:**

Data were sampled from 53 healthcare organizations in 2018. Organizations represented both ambulatory care practices and health systems participating in the state of Kansas HIE. Fourteen ambulatory quality measures for 5300 patients were calculated using the data from an individual EHR source and contrasted to calculations when HIE data were added to locally recorded data.

**Results:**

A total of 79% of patients received care at more than 1 facility during the 2018 calendar year. A total of 12 994 applicable quality measure calculations were compared using data from the originating organization vs longitudinal data from the HIE. A total of 15% of all quality measure calculations changed (*P* < .001) when including HIE data sources, affecting 19% of patients. Changes in quality measure calculations were observed across measures and organizations.

**Discussion:**

These results demonstrate that quality measures calculated using single-site EHR data may be limited by incomplete information. Effective data sharing significantly changes quality calculations, which affect healthcare payments, patient safety, and care quality.

**Conclusions:**

Federal, state, and commercial programs that use quality measurement as part of reimbursement could promote more accurate and representative quality measurement through methods that increase clinical data sharing.

## INTRODUCTION

Clinical quality measures identify opportunities to improve care, report performance, and increasingly contribute to healthcare provider reimbursement.^1–2^ As electronic health record (EHR) adoption has risen dramatically over the past decade, many providers now must generate and submit quality reports through their EHR or another certified technology.^3^^,^^4^ These reports typically use standardized measures, known as electronic clinical quality measures (eCQMs), to report performance to healthcare payers. The largest U.S. program for EHR-based quality reporting is the Merit-based Incentive Payment System (MIPS) that affects Medicare payments. This program attaches up to an 18% annual adjustment to a physician’s payment rate, the largest component of which is quality performance. In 2020, about 890 000 clinicians are subject to MIPS.^5^

### Impediments to EHR quality reporting

Care providers often describe EHR quality reporting as costly, difficult, or irrelevant to their practice given available measures. A 2015 study estimated the cost of quality reporting for general internists, family physicians, cardiologists, and orthopedists at $15.4 billion annually.^6^ Much of this cost stems from time spent documenting care through varying workflows to make quality calculation possible. In 2020, the Centers for Medicare and Medicaid Services (CMS) acknowledged the significant documentation burden resulting from EHR quality reporting.^7^ While EHRs and other technology are required to have the capability to calculate care quality, many are neither easy to use nor liked by clinicians due to concerns about their validity.^2^^,^^8^^,^^9^

The accuracy of EHR-based quality reporting has shown mixed performance when compared with manual chart review.^10^^,^^11^ Electronic quality reporting is frequently inaccurate due to challenges in data completeness, accuracy, terminology use, gaps between structured fields and available free text, and inconsistency of measure logic implementation and certification.^11–14^ Data completeness and interoperability between EHRs are specific concerns given known fragmentation of patient care and data across institutions.^15–17^ For example, EHR problem lists showed significant increases in completeness when clinicians are encouraged to include data from previous encounters and external sources.^18^^,^^19^ When 2 providers see the same patient in a given year, but only one records that the patient is diabetic, diabetes-related eCQMs from those separate EHRs will conflict. Missing diagnoses are one source of variation in quality calculations, and similar discrepancies can be caused by varying completeness and recency of encounter, immunization, medication, laboratory result, procedure and vital sign information. Previous research has shown that data incompleteness and lack of data exchange between systems may impair clinical care, patient safety, and secondary research.^20–24^

### Health information exchanges and quality measurement

Health information exchanges (HIEs) are organizations that facilitate data exchange across multiple institutions, often using different EHRs within a geographical region. There are over 100 HIEs in the United States, many of which collect data in a centralized repository.^25^ These repositories provide a longitudinal data source that may address data incompleteness concerns related to quality measurement and other reporting.^26^^,^^27^ While previous research has explored potential benefits of HIEs on healthcare utilization, costs, and quality, there has been limited research on how data completeness facilitated by an HIE quantitatively affects quality measure calculation.^28–30^

### Study aim

This study examines how electronic clinical quality measurement varies when using data collected by an individual organization vs longitudinal data collected by a statewide HIE operating as a multisource registry for quality measurement. We sought to quantify and characterize the impact of missing data across a range of ambulatory eCQMs and organization types.

## MATERIALS AND METHODS

### Data sources

The Kansas Health Information Network (KHIN) operates a statewide HIE for Kansas, connecting over 10 000 clinicians with approximately 95% of hospitals and 75% of ambulatory providers in the state. Since 2017, KHIN has operated as a clinical registry authorized by CMS and submitted data on behalf of providers for MIPS in 2017, 2018, and 2019.

To exclude facilities that did not regularly contribute data for a majority of the calendar year, we sampled data across all KHIN facilities that had at least 100 clinical documents exchanged monthly for more than 6 months from January to December 2018. Clinical documents were defined as any document using the HL7 (Health Level 7) Clinical Document Architecture standard, the vast majority of which were Continuity of Care Documents.^31^ We grouped 119 facility names meeting these criteria into integrated medical practices or health systems to equally represent organizations that separated specialties and office locations vs those that did not. In addition, 5 facilities not located in Kansas were removed. These steps resulted in 53 distinct organizations.

We randomly sampled 100 distinct patients from each organization in which no inclusion or exclusion criteria were applied (ie, patients may or may not qualify for any quality measure). For the resulting 5300 patients, all KHIN clinical data were extracted from all facilities participating in the HIE, consisting of clinical documents and HL7 messages. To remove duplicate records of the same encounter, only the most recent record from a specific facility on that date was included for each patient. Records were not deduplicated over multiple dates or across facilities before loading into the software for quality measurement.

### Quality measure selection and technology

We selected 14 quality measures that represent a range of preventive care screenings (eg, breast, cervical, and colon cancer screening), measures of disease control (eg, glucose control among diabetic patients, blood pressure control among hypertensive patients), and patient safety (eg, high-risk medication use in the elderly). Because patients were randomly sampled, measure selection also valued variety across age ranges and common clinical conditions. All selected measures are eligible for MIPS eCQM reporting and many have been profiled in previous research.^9^^,^^10^^,^^32–34^

The software (Diameter Health version 3.59; Diameter Health, Farmington, CT) used for electronic quality measurement has been demonstrated in prior research and is certified by the National Committee for Quality Assurance and the Office of the National Coordinator for Health Information Technology.^32^ Certification allows for the use of this technology in MIPS and other quality reporting programs. The technology has been used by KHIN and other organizations since 2017.

### Quality measure comparison

Each extracted record was categorized as either originating from the organization where the patient was sampled (ie, the originating organization) or from another KHIN data source. This allowed for quality measurements to be calculated twice. The first calculations included data only from the originating organization for all 5300 sampled patients. The second calculations included data from the originating organization and other KHIN data sources for these same 5300 patients.

Quality measures were calculated by patient and then aggregated by organization and measure to total the number of discrepancies and calculate performance rates. In calculations designated as not applicable, the measure eligibility criteria (ie, denominator logic) were not fulfilled. For calculations meeting eligibility criteria (ie, applicable quality measures), a patient outcome was characterized as excluded if the patient met exclusionary criteria for the measure, or clinically compliant or noncompliant if no exclusion criteria were met, in which compliant represents recommended care.

### Data presentation and statistical analysis

Patient demographics, record counts, and applicable quality measures were summarized by organization type (health system vs ambulatory practice). For all applicable measures, we examined quality measure calculation changes based on the inclusion of all HIE data compared with the originating organization only. While both calculations would be eligible for value-based quality reporting affecting provider payments, we consider the usage of all available data to be the reference standard because it incorporates more complete and longitudinal data from the HIE.

Pairwise changes for each applicable quality measure by patient were summarized and statistically examined using McNemar’s test, adapted for a multinomial extension for the 4 outcomes of quality measurement (ie, not applicable, excluded, noncompliant, or compliant). Each of the 14 measures was also examined independently for statistical significance using a Monte Carlo multinomial test corrected for false discovery rate. Quality measure discrepancies were examined by facility to determine whether quality measure changes were localized or broadly observed.

## RESULTS

### Facilities and data sampled

Of the 21 health systems sampled, 10 were critical access hospitals and 11 were prospective payment hospital-based health systems. Of the 32 ambulatory care practices, 21 were private practices, 8 were federally qualified health centers, and 3 were mental health centers. The sampled facilities used 15 different EHR technologies, including several major vendors such as Allscripts (Chicago, IL), Cerner (Kansas City, MO), eClinicalWorks (Westborough, MA), Epic (Verona, WI), Meditech (Westwood, MA), and NextGen (Irvine, CA).

### Record distribution and quality calculation

Of the 5300 patients sampled for quality measure calculation, 5 were rejected by the software due to large clinical record size (mean = 989 MB). Of the remaining 5295 patients with quality calculations, 2100 (38.9%) were sampled from integrated health systems and 3195 (61.1%) were sampled from ambulatory practices. Because patients were randomly selected based on data presence reflecting healthcare received across the HIE, their distribution skews older (mean = 46.5 years of age) and more female (59.6%) than the overall Kansas population.^35^ Nonetheless, the sample did represent a mix of patients across multiple age, race, and ethnicity categorizations as shown in Table 1.

**Table 1.** Demographics and Data Distributions of Sampled Patients

	Sampled Facilities	
	Health Systems	Ambulatory Practices
**Data sampled**		
Facilities	21	32
Patients	2100	3195
Average records per patient^a^	21.35	19.87
**Sex**		
Female	57.0%	61.3%
Male	43.0%	38.7%
**Age**		
0-4 y	7.5%	5.8%
5-17 y	9.4%	12.7%
18-64 y	48.5%	57.3%
65+ y	34.6%	24.2%
**Race**		
White	84.9%	76.8%
Black	4.3%	6.7%
Other or unknown	10.8%	16.6%
**Ethnicity**		
Non-Hispanic	90.5%	92.8%
Hispanic	9.5%	7.2%
**Quality measures calculated**		
Mean applicable quality measures per patient	2.44	2.39
Fraction of patients with 1 or more applicable quality measures	87.1%	82.5%

a Records were HL7 (Health Level 7) formatted clinical documents or sets of HL7 messages.

For the patients sampled, 1712 (81.5%) from health systems and 2456 (76.9%) from ambulatory practices had data from sources besides the originating organization. In total, 50 903 (46.4%) of the 109 805 total records came from sources other than the originating organization, effectively doubling the data used in quality calculations when including HIE data.

**Figure 1.**
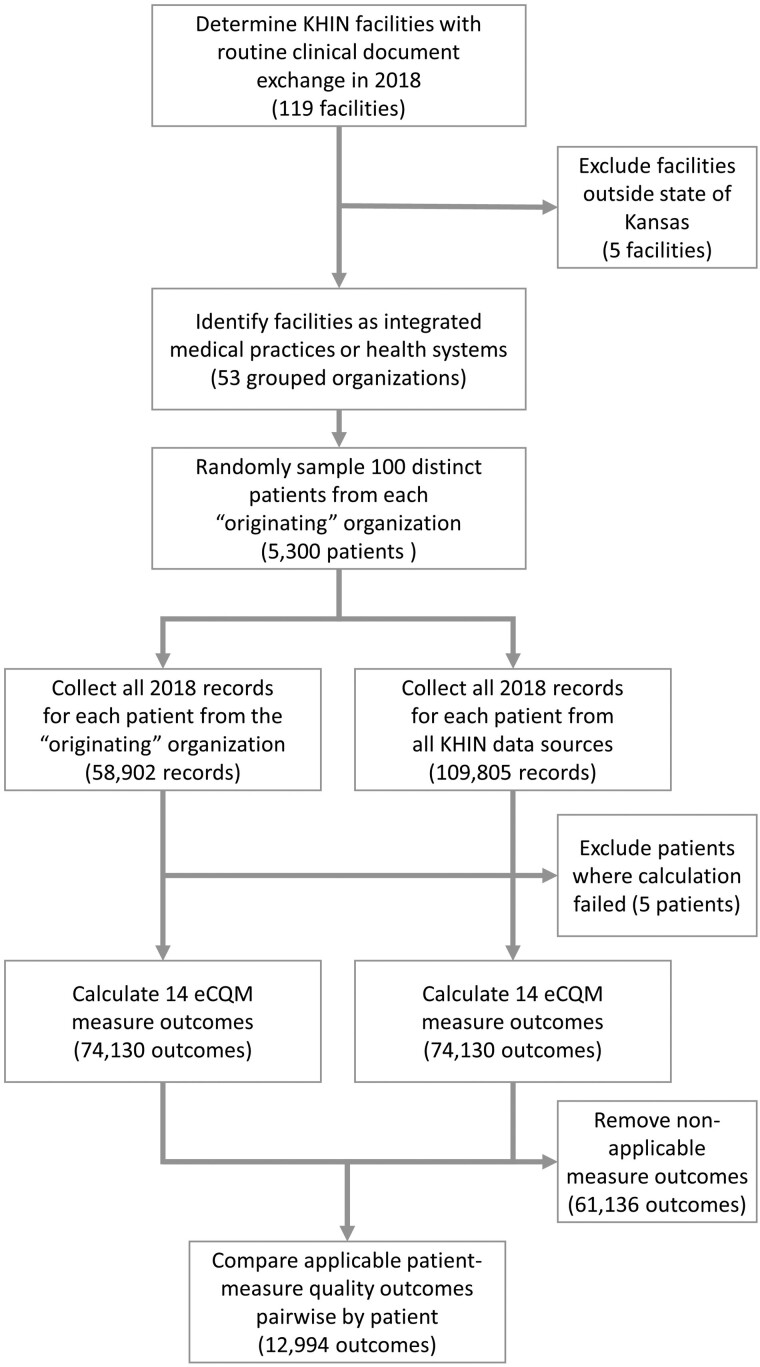
Flow diagram of patient selection and quality calculation. eCQM: electronic clinical quality measure; KHIN: Kansas Health Information Network.

Not every quality measure was applicable to every patient due to age, sex, and disease inclusion criteria. A total of 4466 (84.3%) patients qualified for at least 1 quality measure, with the mean applicable quality measures per patient being 2.4 ±2.2 (range, 0-9). The patient selection and quality calculation process are shown as a flow diagram in Figure 1. In total, there were 12 994 applicable quality measure calculations that could be compared between the 2 methods for quality measurement.

### Quality measure comparison

**Figure 2.**
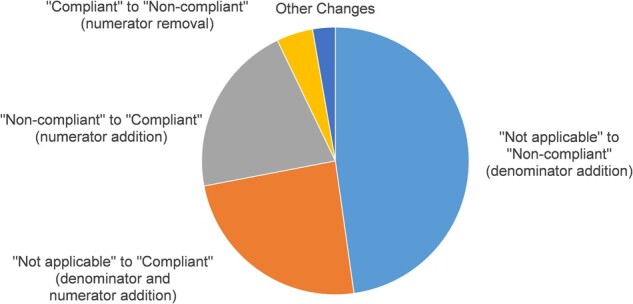
Discrepancies in quality measure calculations.

Each of the 12 994 measurements was compared between the calculations for originating organization data and all KHIN data. A total of 1974 (15.2%) of these measurements changed when all data were included (*P* < .001). The types of these discrepancies in quality measurement are shown in Figure 2. The largest discrepancies were changes from not applicable to noncompliant (n = 943) and not applicable to compliant (n = 478), which occurred when a patient became eligible for a measure through inclusion of additional data from the HIE. The next largest change was from noncompliant to compliant (n = 412), which occurred when data recorded elsewhere in the HIE enabled the patient to achieve compliance. The last major discrepancy was from compliant to noncompliant (n = 87), which occurred when more recent and complete information overrode compliance that would have been measured by the EHR data alone. Overall, 1000 (18.9%) patients were subject to at least 1 change in quality measure calculation.

Significant differences in quality measure calculations were observed for 13 of the 14 measures (*P* < .001). The one measure that did not show significance was the episodic measure of appropriate testing for childhood pharyngitis. Changes in individual quality measure calculations may or may not affect overall quality performance rates. For example, if 10 hypertensive patients are found to have an increase in blood pressure before end of year, while 10 are found to have a decrease (ie, 20 discrepancies), there would be no net change to the practice’s reported compliance. To determine if overall compliance was affected by individual discrepancies, we examined compliance change by measure. The measure for high-risk medication use in older adults showed a decrease in compliance. Nine other measures, mostly related to preventive care or disease management, showed an increase. Four measures showed minimal (<1.0%) absolute change in compliance. Overall changes in compliance as well as the types of measure discrepancies are shown in Table 2.

**Table 2.** **Impact to Quality Measures Based on** Health Information Exchange **Data Inclusion**

	Compliance Change	Applicable Calculations	Quality Measure Discrepancies					
			NA to NC	NA to CC	NC to CC	CC to NC	Other	Significance (*P*)
**Measures with negative change in compliance**								
High risk medication use in older adults (cms156)	−4.6%	1374	3	51	3	68	0	<.001
**Measures with minimal change in compliance**								
Diabetes: annual eye exam (cms131)	−0.1%	776	132	0	1	0	0	<.001
Primary caries prevention (cms74)	−0.1%	806	166	0	0	0	0	<.001
Diabetes: annual foot exam (cms123)	−0.1%	776	131	0	0	0	2	<.001
Appropriate testing for children with pharyngitis (cms146)	0.3%	21	3	1	0	0	0	.0512
**Measures with positive change in compliance**								
Breast cancer screening (cms125)	1.2%	1017	49	3	13	0	6	<.001
Chlamydia screening for women (cms153)	2.6%	111	14	4	2	0	0	<.001
Cervical cancer screening (cms124)	2.9%	1517	81	8	40	0	7	<.001
Colorectal cancer screening (cms130)	3.1%	1856	72	12	49	0	11	<.001
Controlling high blood pressure (cms165)	4.8%	1220	98	137	56	18	28	<.001
Pediatric weight assessment: BMI percentile (cms155)	6.4%	509	41	93	13	0	0	<.001
Pneumonia vaccination of older adults (cms127)	7.0%	1459	38	20	94	0	0	<.001
Diabetes: poor HbA1c control (cms122)	10.7%	776	85	47	80	1	0	<.001
Diabetes: attention for nephropathy (cms134)	10.8%	776	30	102	61	0	0	<.001
**Total across 14 ambulatory measures**		**12 994**	**943**	**478**	**412**	**87**	**54**	<.001

Full measure definitions for each eCQM available at https://ecqi.healthit.gov.

BMI, body mass index; CC: clinically compliant; eCQM: electronic clinical quality measure; NA: not applicable; NC: noncompliant.

### Facility distribution

We found a wide variance on the impact of quality measurement by organization. Overall, 42 of the 53 organizations had more than 5% of their quality measure calculations change based on the inclusion of data from the HIE. Four ambulatory practices had 100% of their calculations change. For these facilities, further investigation revealed that they were not recording encounter information in an appropriate format and did not have any eligible calculations before the inclusion of other facility data; therefore, quality measures could only calculate when other facility data were included.

**Figure 3.**
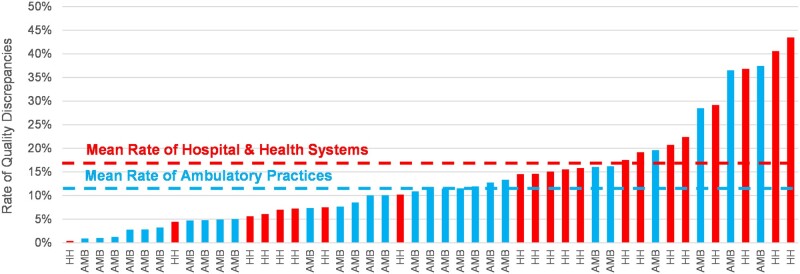
Rate of quality measure discrepancies by facility. Hospital or health system (HH) is shown in red and ambulatory practice (AMB) is shown in blue.

Figure 3 displays the overall rate of quality measure variance for health systems and ambulatory practices, excluding the facilities with 100% discrepancy rates. The mean rate of quality measure discrepancies was 16.5 ± 12.0% for health systems and 11.2 ± 9.3% for ambulatory practices.

## DISCUSSION

Previous research has demonstrated that patient care is fragmented and a single EHR may omit relevant clinical information.^16^^,^^19^^,^^27^^,^^28^ This study demonstrates that adding standards-based data available through an HIE significantly changes quality measurement. Including longitudinal data often results in performance rate improvements, although any change can be viewed as more complete measure calculation that includes all relevant data. Reviewing these improvements and interoperability’s role in quality calculation contextualize the impact to efficient care, patient safety, and value-based payment programs. Policy implications from these findings have the potential to improve the accuracy and robustness of future quality measurement.

### Data incompleteness leads to quality measurement discrepancies

Previous studies have demonstrated that EHR data are often incomplete, although the extent to which this affects quality measurement has not been directly examined.^17–19^^,^^22–24^ One prior study demonstrated that external data access was correlated with quality measure improvements but compared different organizations managing varying patient populations.^30^ Our current cross-sectional study examines the same patients and organizations with and without longitudinal data inclusion and demonstrates that 15% of quality measure calculations, affecting nearly one-fifth of sampled patients, change when longitudinal data from an HIE are included. Quality measure discrepancies range across different types of quality measures and organizations and provide additional evidence that external data access can affect quality measurement.

The largest source of quality measure discrepancies are inclusion criteria, which rely on eligible encounters and documented conditions. Prior research has shown EHR problem list incompleteness rates between 10% and 60% for an individual health system.^18^^,^^19^^,^^23^ For the 4 measures that included diagnoses as part of the measure calculation (ie, diabetes and hypertension measures), over 24% of measure calculations change when HIE data are included. Because data sharing through an HIE closes a portion of incomplete diagnoses before quality calculation, the magnitude of measure discrepancies observed in this research generally align with incompleteness expectations from other research.

This research demonstrates that discrepancies affect performance rates for some, but not all, quality measures. For example with glucose control among diabetic patients (ie, cms122), a 10.7% performance variance improves relative performance by more than 2 deciles based on 2019 benchmarks.^33^ While most measures show favorable changes in compliance, the use of high-risk medications in older adults (ie, cms156) shows a 4.6% decrease. This variance could move an organization from the 90th to the 30th percentile of performance based on 2019 benchmarks.^33^ While changes in relative performance are important because they impact payments associated with value-based care programs, any quality measure discrepancy matters in context of the patient. Knowing patient measure eligibility informs care guidelines and clinician outreach, and knowing patient compliance reduces unnecessary communication and duplicate testing. Many studies have examined how HIEs can contribute to reduced cost and duplicate testing, and this study supports that more longitudinal quality measurement is another opportunity for organizations facilitating clinical data sharing.^28–30^

### Impact on care efficiency, patient safety, and payment

The time and costs associated with quality reporting are significant and many clinicians express frustration with the process.^6^^,^^9^ This study’s methods perform quality measurement based on standards-based data exchange and incur no incremental time or effort on the behalf of sampled facilities. Data already exchanged using clinical data standards allow for calculation of many quality measures.^32^ While the primary intent of this research was to determine whether there was a significant difference in quality measurement when longitudinal data from an HIE were included, this approach has the potential to reduce administrative effort and decouple eCQM calculation from any specific EHR. While not directly measured in this research, lack of relevant measures in an EHR has been a documented concern.^8^^,^^9^ The methods employed in this research support ongoing initiatives to integrate interoperability standards into the quality measurement process.^15^^,^^36^^,^^37^

In addition, data incompleteness has a role in patient safety.^38^ This study found that data sharing identifies high-risk medication use (cms156) that may not have been detected without clinical data exchange. This measure draws on consensus guidance from the American Geriatrics Society and research connecting specific drugs to preventable adverse drug events, poorer health status, and increased risk of death.^39–41^ The 68 measure changes from compliance to noncompliance point to individual organizations having incomplete medication histories and illustrate how data sharing affects both patient safety and quality measurement.

Ambulatory quality performance rates also affect payments for clinicians that participate in Medicare, state, and commercial value-based contracting. Performance rates that impact a meaningful portion of provider payments therefore should be highly vetted and rigorous. Calculating how clinical data sharing impacts specific payments was not performed in this research because insurance mix affects value-based payments and clinicians may report measures other than those examined. Comparing observed performance changes to relative benchmarks, however, suggests that measurement changes are likely important for some measures. Measurement discrepancies contributed roughly equally to noncompliant and compliant outcomes (inaccuracies of 1030 and 890, respectively), although a majority of measures showed a positive change in reported compliance, as shown in Table 2.

### Interoperability and quality measurement

Interoperability and quality measurement are often considered separate initiatives in the scope of healthcare operations, although their interrelationship has increasingly been recognized.^15^^,^^36^^,^^37^ This research demonstrates that clinical data sharing significantly affects quality measurement. It also substantiates findings by CMS and others that federal programs for EHR adoption have not achieved widespread interoperability.^38^^,^^42^ While EHRs remain a primary source of clinical quality calculations today, research supports that EHRs and associated workflow challenges often result in inadequate data for accurate quality measure calculations.^9^^,^^12^ This study supports that alternative infrastructures for more accurate quality calculation may be possible.

This study also provides evidence that EHR-based eCQM reporting discrepancies are not isolated occurrences because variation was observed across many organizations. In this research, the automated inclusion of HIE data provides a reference standard for longitudinal eCQM calculation. In contrast to eCQM programs, the Health Effectiveness Data and Information Set (HEDIS), which is the longest established program for U.S. quality measurement by health plans, always bases its program on multisource, longitudinal billing data for a patient. Although using billing data, as opposed to clinical EHR data, has some downsides (eg, the presence of a lab test is detectable but its value may not be), it has the advantage that if any provider records eligible diagnoses or procedures, then they apply universally to quality measurement for that patient. This minimizes errors due to data incompleteness, as partly reflected through national benchmark data between HEDIS and eCQM programs. For example, the average rate of colorectal cancer screening is 65% to 75% nationally based on the HEDIS program and 71% according to CMS claims-based reporting.^33^^,^^43^ The eCQM-based performance rate for this measure collected through MIPS is substantially lower at 44%.^33^ While KHIN does not include claims data in its centralized repository, other HIEs do, and prior research suggests that billing data integration may improve quality measurement.^34^^,^^44^ Future research should explore how data aggregation that incorporates both longitudinal clinical and claims information may improve quality measurement.

### Policy implications

Three policy implications emerge from an acknowledgment that individual EHRs often have incomplete data for quality measurement. First, ambulatory quality programs that encourage data interoperability may improve quality calculation for practices and health systems. Health plans have long appreciated that HEDIS measures are computed across all the patient’s care settings and invest heavily in data completeness. Healthcare providers burdened with interoperability challenges and limited resources may not obtain patients’ medical records from disparate providers to ensure complete information for accurate eCQM calculation.^36^ Refocusing quality programs on data sharing reinforces the value of data interoperability and reduces the provider pressure to chase medical records from all possible locations.

Next, harmonized quality reporting methods can provide more consistency in quality measurement. While some facilities saw measure discrepancy rates over 20% in this research, others were below 5%. Until widespread interoperability is achieved, longitudinal calculation or EHR-based episodic quality measurement may provide more comparable performance rates.

Finally, effective data sharing provides an alternative infrastructure for measure calculation. This has the potential to reduce administrative burden, cost, and frustration associated with measure calculation. Longitudinal data aggregation using standards, such as those promoted by the Office of the National Coordinator for Health Information Technology and National Committee for Quality Assurance, can provide a foundation for this transition. This was demonstrated at a statewide HIE in this research, although other data aggregators could be explored in future research.^37^ While not all measures work equally well through standard-based automated measure calculation, pairing interoperability with quality measurement may benefit patient safety, streamlined calculation, and measurement accuracy.^32^^,^^37^^,^^38^^,^^45^

### Limitations

This research has several limitations based on data availability and study methods. Although most providers in Kansas participate in KHIN, data sharing remains a voluntary process. Therefore, standards-based exchange used in this research may still have data gaps that affect quality measurement. While electronic quality measurement using certified technology as performed in this research affects provider payments today, the reference standard of longitudinal data inclusion may vary from manual chart abstraction and methods incorporating billing information. In addition, this study’s patient selection process of random patient selection at healthcare facilities biases results toward patients more likely to pursue care. Most eCQM programs implicitly include this bias, while other programs like HEDIS examine all health plan members.

This study focused on 14 ambulatory quality measures using certified technology, which represent a fraction of the over 200 measures available for MIPS ambulatory quality reporting.^33^ Future studies could examine the impact on other measures as well as quantify data incompleteness independent of quality measurement. Finally, quality improvement and reporting initiatives of the selected facilities were not taken into consideration. While having a large set of organizations increases the generalizability of our findings, focused quality improvement efforts may affect the magnitude of observed changes for an individual institution.

## CONCLUSION

Electronic quality reporting has been required as a part of EHR adoption over the past decade. While certified EHRs have the capability to calculate quality measures as required by MIPS, individual EHR-based calculations differ significantly when compared with multisource, longitudinal calculations. While this is a natural consequence of patients seeing multiple providers annually and incomplete interoperability, this study finds that data sharing affects patient safety and measures routinely used in value-based payment models. Therefore, programs that incorporate longitudinal data are more likely to result in accurate quality measurement. Federal policies that promote data interoperability and the harmonization of reporting methods may result in more accurate and representative quality measurement in the coming years.

## FUNDING

This work was supported by Kansas Health Information Network and Diameter Health that jointly donated time and resources to the research team.

## AUTHOR CONTRIBUTIONS

All members of the research team were needed for various portions of the design, research, analysis, and writing and the safeguarding of protected health information. All contributing authors were involved with the following areas: substantial contributions to the conception and design of the work; the acquisition, analysis, or interpretation of data for the work; drafting the work or revising it critically for important intellectual content; final approval of the version to be published; agreement to be accountable for all aspects of the work in ensuring that questions related to the accuracy or integrity of any part of the work are appropriately investigated and resolved.

## ETHICS APPROVAL

This study was approved by the Institutional Review Board for of the University of Texas Health Science Center at Houston’s Committee for the Protection of Human Subjects. Technical and administrative safeguards were utilized to protect the privacy of such information throughout this research.

## CONFLICT OF INTEREST STATEMENT

JDD, CL, PT, and CJV receive salaries from and have an equity interest in Diameter Health, Inc., whose software provided the quality measure calculation used in this research. DFS serves as a scientific advisor with an equity interest in Diameter Health.

## DATA AVAILABILITY

The primary data underlying this article cannot be shared publicly due to the privacy of patients within the Kansas Health Information Network. Aggregate, de-identified data will be shared on reasonable request to the corresponding author. Benchmark data used in this research from federal quality payment programs are available through public websites (https://github.com/CMSgov/qpp-measures-data, https://www.ncqa.org/hedis/measures/).
